# SMS feedback system as a quality assurance mechanism: experience from a household survey in rural India

**DOI:** 10.1136/bmjgh-2021-005287

**Published:** 2021-07-21

**Authors:** Neha Shah, Osama Ummer, Kerry Scott, Jean Juste Harrisson Bashingwa, Nehru Penugonda, Arpita Chakraborty, Agrima Sahore, Diwakar Mohan, Amnesty Elizabeth LeFevre, Kilkari Impact Evaluation Team, Smisha Agarwal

**Affiliations:** 1 International Health, Johns Hopkins University Bloomberg School of Public Health, Baltimore, Maryland, USA; 2 Oxford Policy Management-Delhi, New Delhi, Delhi, India; 3 Independent Researcher, Bangalore, Karnataka, India; 4 Computational Biology Division, Department of Integrative Biomedical Sciences, Institute of Infectious Disease and Molecular Medicine (IDM), Faculty of Health Sciences, University of Cape Town, Cape Town, Western Cape, South Africa; 5 BeeHyv Software Solutions, Raja Praasadamu, Hyderabad, Telangana, India; 6 School of Public Health and Family Medicine, University of Cape Town, Cape Town, Western Cape, South Africa

**Keywords:** qualitative study, public health

## Abstract

The increasing use of digital health solutions to support data capture both as part of routine delivery of health services and through special surveys presents unique opportunities to enhance quality assurance measures. This study aims to demonstrate the feasibility and acceptability of using back-end data analytics and machine learning to identify impediments in data quality and feedback issues requiring follow-up to field teams using automated short messaging service (SMS) text messages. Data were collected as part of a postpartum women’s survey (n=5095) in four districts of Madhya Pradesh, India, from October 2019 to February 2020. SMSs on common errors found in the data were sent to supervisors and coordinators. Before/after differences in time to correction of errors were examined, and qualitative interviews conducted with supervisors, coordinators, and enumerators. Study activities resulted in declines in the average number of errors per week after the implementation of automated feedback loops. Supervisors and coordinators found the direct format, complete information, and automated nature of feedback convenient to work with and valued the more rapid notification of errors. However, coordinators and supervisors reported preferring group WhatsApp messages as compared with individual SMSs to each supervisor/coordinator. In contrast, enumerators preferred the SMS system over in-person group meetings where data quality impediments were discussed. This study demonstrates that automated SMS feedback loops can be used to enhance survey data quality at minimal cost. Testing is needed among data capture applications in use by frontline health workers in India and elsewhere globally.

**Summary box**

- The quality of household survey data depends on both intrinsic and extrinsic factors, and technology can play a role in enhancing quality assurance measures.
- Implementing a short messaging service (SMS)-based quality assurance programme for field staff completing a household survey was perceived positively by the field team and reduced error resolution time.
- The SMS error messages included all the key information required to resolve errors without relying on group meetings or additional information sent manually and were more timely than manual notifications.
- The SMS modality was acceptable, but the field team preferred WhatsApp over SMS, and they also preferred group rather than individual messages because they discussed and sent corrections via WhatsApp group messages.
- Future surveys may implement a SMS feedback system considering differences in modality, recipients, and timeliness.

## Background

Data on the utilisation of health services, health behaviours, and health status of the population is vital for informing decision-making on the optimal amount and allocation of resources. In many low-middle-income countries, these data often come from wide-ranging household surveys or increasingly, digital health data capture applications. The effective use of these data depends heavily on the quality of data.^1^ Most approaches to monitoring data quality have focused on regulating intrinsic factors, such as the tool itself and the system underlying its use. Extrinsic factors include the location, setting, community environment, and enumerator–respondent dynamics, impacting survey implementation and the resulting data quality. Data quality is shaped at its core by the structure and quality of the survey tool itself including the indicators selected, language used, and the nature of the response options. Beyond the tool, data quality is impacted by the implementation of the tool including the modality (digital tool vs. paper, face-to-face vs. phone survey) and location of implementation; the profile, number, training, and supervision of enumerators; and routine monitoring of incoming data.^1^

India is home to some of the largest digital data capture applications globally both with regard to routine data capture by frontline health workers and in the context of special surveys. Some applications include Integrated Child Development Services-Common Application Software^2^ used by Anganwadi workers and Auxiliary nurse midwife online (ANMOL),^3^ which is used by auxiliary nurse midwives. The Indian Comprehensive National Nutrition Survey uses short messaging service (SMS) notifications to monitor the quality of biomedical samples collected in the field.^4^

The widespread availability and use of technology including tablets and mobile phones, for collecting health data as well as coordinating logistics among supervisors and enumerators, has widened the possibilities of technology use for improving data quality assurance (QA) measures. QA includes those activities that ensure the data are of high quality such as documentation, checks on the data, and adequate reporting.^5^ Technology use during data collection has also been shown to reduce costs and increase efficiency.^6^ Technology use for data QA has the potential to expand beyond data captured to additionally harness the use of mobile phones used for communication by those capturing data (survey enumerators, healthcare providers) and their supervisors.

To date, SMS text messages have been used as a communication modality between healthcare providers and seekers for healthcare reminders,^7^ and adherence to medications^7 8^ or immunisation schedules.^9^ The same technology, linked with efforts to identify and automate data capture quality impediments could be implemented to improve QA as part of routine data capture applications or surveys.

In this analysis, we outline findings from efforts in four districts of Madhya Pradesh, India, to improve the quality of household survey data for the evaluation of a maternal-mobile messaging programme.^10^ We sought to improve quality during implementation through the use of automating identification of errors and feeding these errors back to supervisors in near-real time using SMS text messages. We start by reviewing routine QA procedures and developing a system to enhance survey QA methods. We then examined the perceptions of this system and timeliness of this system as compared with manual notification of errors.

## Establishing an enhanced QA system

We manually tracked major errors at the beginning of data collection in October 2019 (table 1). For every error identified, we collected: the respondent unique identifier, date of interview, supervisor identification, enumerator identification and error field. This information was stored in a google spreadsheet which was then shared with the survey coordinator. The survey coordinator held weekly in-person meetings with the field team. In these meetings, they resolved errors after supervisors and enumerators either checked their notes or called the respondent.

**Table 1:** Description of errors tracked and sent to field staff

Short messaging service error message	Error definition
Duplicate unique identifier	A 15-digit unique identifier duplicates another unique identifier in the data; the identifier is a concatenation of the district code, block code, village code, household number, and structure number.
Incorrect birthdate	Child’s birthdate, including date, month, and year, has been incorrectly entered. Birthdate is too recent (less than 11 months since birth) or too old (more than 17 months since birth).
Many miscarriages	More than one miscarriage noted for one pregnancy event.
Many births	More than two births noted for one pregnancy event.
Young age	Respondent reported age is 18 or below.
Female condoms	Female condoms use reported despite the fact that they are rare in rural India.
Rushed interviews	Surveys taking an abnormally short time to complete based on a machine learning algorithm.

**Figure 1:**
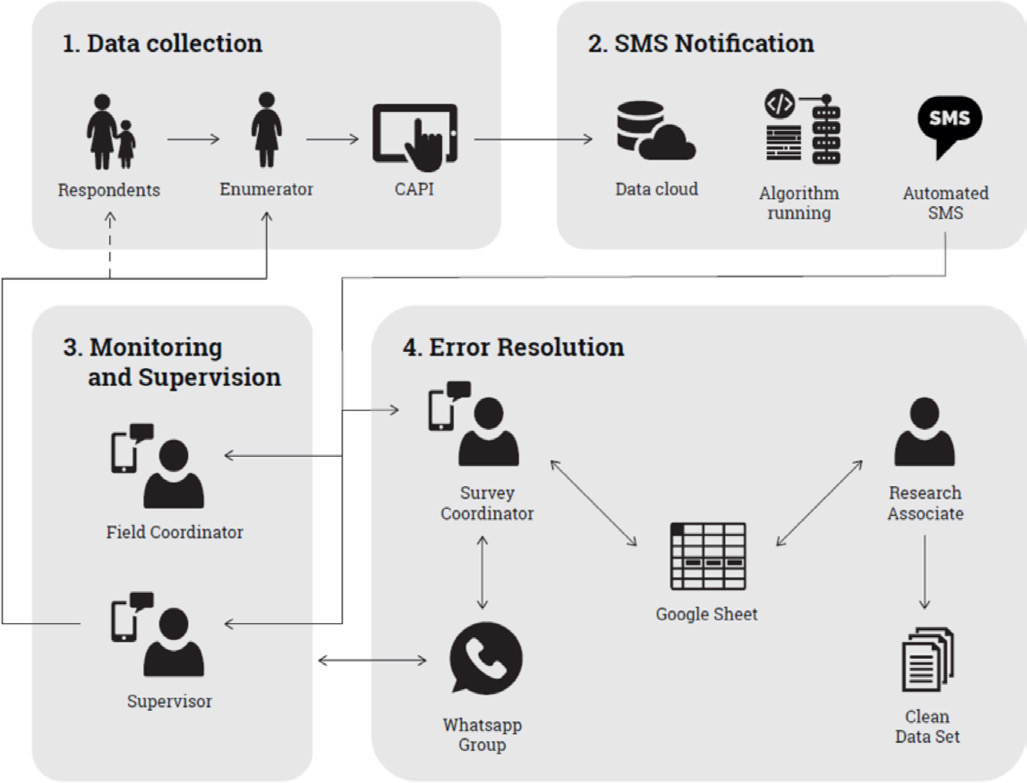
Framework of quality assurance data flow. CAPI, Computer-Assisted Personal Interviewing; SMS, short messaging service.

An automated SMS notification system was implemented in early February 2020, 16 weeks into data collection. This SMS feedback system was implemented based on the fact that survey data were collected on Computer-Assisted Personal Interviewing (CAPI). CAPI allows for real-time data collection, automatic skip patterns, and embedded limit-checks, which allows us to both limit the number of errors, but also identify any further errors soon after the data are collected. This SMS system used a transactional account through the Textlocal company to send out the SMSs. We applied machine learning using extreme gradient boosting to tag interviews that were completed too fast. Gradient boosting machines are powerful ensemble machine learning algorithms that use decision trees. The data set we used to model rushed interviews was a set of surveys we asked the enumerators to self-fill. These surveys were completed by the enumerators as fast as they could (without a respondent), resulting in 201 self-filled surveys. The collected time stamps for each section were then processed to determine ‘short’ lengths for each section. We chose the gradient boosted model because it had the greatest sensitivity and specificity of the models we ran.^11^ We automated the code that identified manual errors and rushed interviews using the Linux crontab command. The code that identified manual error and trained the model were constantly deployed on new data sets uploaded to a shared Dropbox, and SMSs were sent out the evening after the upload. The code outputted the same information as the error log Google sheet for each error SMS. This report was designed to fit the 160-character limit of an SMS. SMSs were sent to the supervisors whose enumerators made errors. Individual enumerators did not receive SMSs. All the error SMSs were sent to the two field coordinators and the survey coordinator. The supervisors were expected to address the SMS notification messages relevant to surveys completed by their team. The errors were also tracked in the Google spreadsheet, but instead of in-person team meetings, supervisors and enumerators consulted with each other when errors arose and sent the corrected values to the survey coordinator via WhatsApp message. The information flow for this system is shown in figure 1. Further methods to analyse this system are listed box 1.

Box 1 **Summary methods**

**Study description:** Examining the perceptions and timeliness of a short messaging service feedback system on data quality assurance in a household survey in Madhya Pradesh, India. The survey was conducted using computer-assisted personal interviewing using tablets.

**Profile of data collectors:** The survey team for the endline surveys consisted of the survey coordinator (n=1), field coordinators (n=2), supervisors (n=9), and enumerators (n=54). Each supervisor managed a field team of approximately six enumerators and travelled with them to oversee data collection activities each day. The survey coordinator, field coordinator, and 8 of the 9 supervisors were all men; 18 of the enumerators were men and 36 were women. All the members of the field team had graduate-level education with previous experience on large surveys.

**Data collection:** Analysis findings draw from two sources of data: (1) error resolution timeliness and (2) qualitative interviews with the field team. Qualitative interviews, including six in-depth interviews and one focus group discussion, were carried out by two social scientists. The in-depth interviews were conducted with the two coordinators, three supervisors who were selected because they received a high number of error messages, and one supervisor who was selected because he received a low number of error messages. The focus-group- discussion included female enumerators from different supervisors’ teams. Qualitative data were analysed using the Dedoose application and quantitative data were analysed using the R statistical package.

**Analyses:** The timeliness of error resolution analysis was based on tracking interview dates and date of error resolution in the google spreadsheet. Qualitative interviews were audio recorded and translated into English transcripts for coding. The coding framework was developed based on a priori domains of interest.

## Existing data quality mechanism

The three existing data quality systems are spot check, 10% recheck, and random check. Spot checks involved the coordinator or supervisors observing parts of interviews based on a standardised form to assess if they were completed with the correct techniques and in the correct conditions. The coordinator or supervisor was expected to submit three completed spot check forms per day. Ten percent recheck involved 10% of respondents interviewed until being re-interviewed by a different enumerator using a random sample of 10–20 questions provided by CAPI. Random check included coordinators and supervisors checking several questions they chose at random by re-asking the respondent those questions before submitting the data to the server.

R: We used to see how they were talking, the way of sitting, if there is any eye contact or not, if privacy has been taken into consideration or not, we used to see all these things. The module where we reached was spot-checked using the spot check form. (SUP_02)

Before the SMS feedback system was put in place, the research associate identified errors via a manual tracking process, and these errors were shared with the survey coordinator, who held weekly meetings with enumerators to resolve the errors. The error log was updated within 1–2 weeks after the interview was completed. Errors were resolved by looking back at field notes, calling the respondent or conferring with other team members during the weekly meetings. The coordinators and supervisors used a WhatsApp group to discuss any data quality issues when not meeting in person.

## Experience with the SMS feedback system

### Content

The field team reported that the SMS content was easy to understand and had all the necessary details to identify the errors and resolve them. The SMSs improved quality checking processes because the concerned supervisors and coordinators could individually contact the enumerator involved rather than calling in-person group meetings. The coordinators felt that the SMSs were more systematic, easier to resolve and reduced their workload because the supervisors also received the SMSs. The enumerators preferred the automatic generation of errors and did not feel that it was impersonal; in fact, they felt that the computer-generated errors would not be biased or unfair. Also, the direct identification of supervisors/enumerators in the error messages meant they were cognizant of their errors and improved based on feedback. The field team said a limitation of this programme was the automated error identification algorithm could sometimes flag correct data as an error. For example, one coordinator mentioned that because the errors are based on upper or lower limits, sometimes the message flags an issue that may no longer be an issue. In the case of eligible birthdates, children 17 months or older were flagged, but towards the end of the data collection many women who were not previously available were interviewed, and thus had older children, but the system initially still flagged them as an issue.

R: In a computer barrier has been put that if the value is above this then a message has to be sent. If a person is sitting and watching, then he can understand. (COR_02)

### Timeliness

Respondents felt that the SMS notification system had made error resolution faster compared with the earlier in-person meetings. Additionally, with the SMS system, they knew the corrections to the errors were relayed back in a timely manner; with the earlier manual system, sometimes corrections were forgotten and not updated.

R: The workload used to increase, and so did the corrections. But when SMS came, we felt better when the work was completed quickly, we didn’t have much tension, and the supervisor also did corrections. (COR_01)

The SMS feedback system was introduced in the last two months of data collection. While enthusiastic about using the system for future surveys, the coordinator did mention early implementation of this system would be beneficial. Another supervisor recommended that SMS arrive at a fixed time in the evening, so the field team can be prepared to discuss. Another suggested daily SMS error notifications rather than twice a week to reduce the workload.

R: Getting daily is better. If we will store it for a week, then the burden will increase. Workload will increase. Today’s work should be completed today only. (SUP_04)

This system requires time to set up because at the beginning of data collection, it is needed to identify the major errors. But during that stage, we also identified errors that were resolved with feedback to the team. In future surveys, we would complete a more rigorous process to identify potential errors before the survey began and identify errors at a faster rate at the beginning of data collection so that we could begin the process of automating error identification sooner and have the SMS feedback system in place in an earlier stage of data collection.

**Figure 2:**
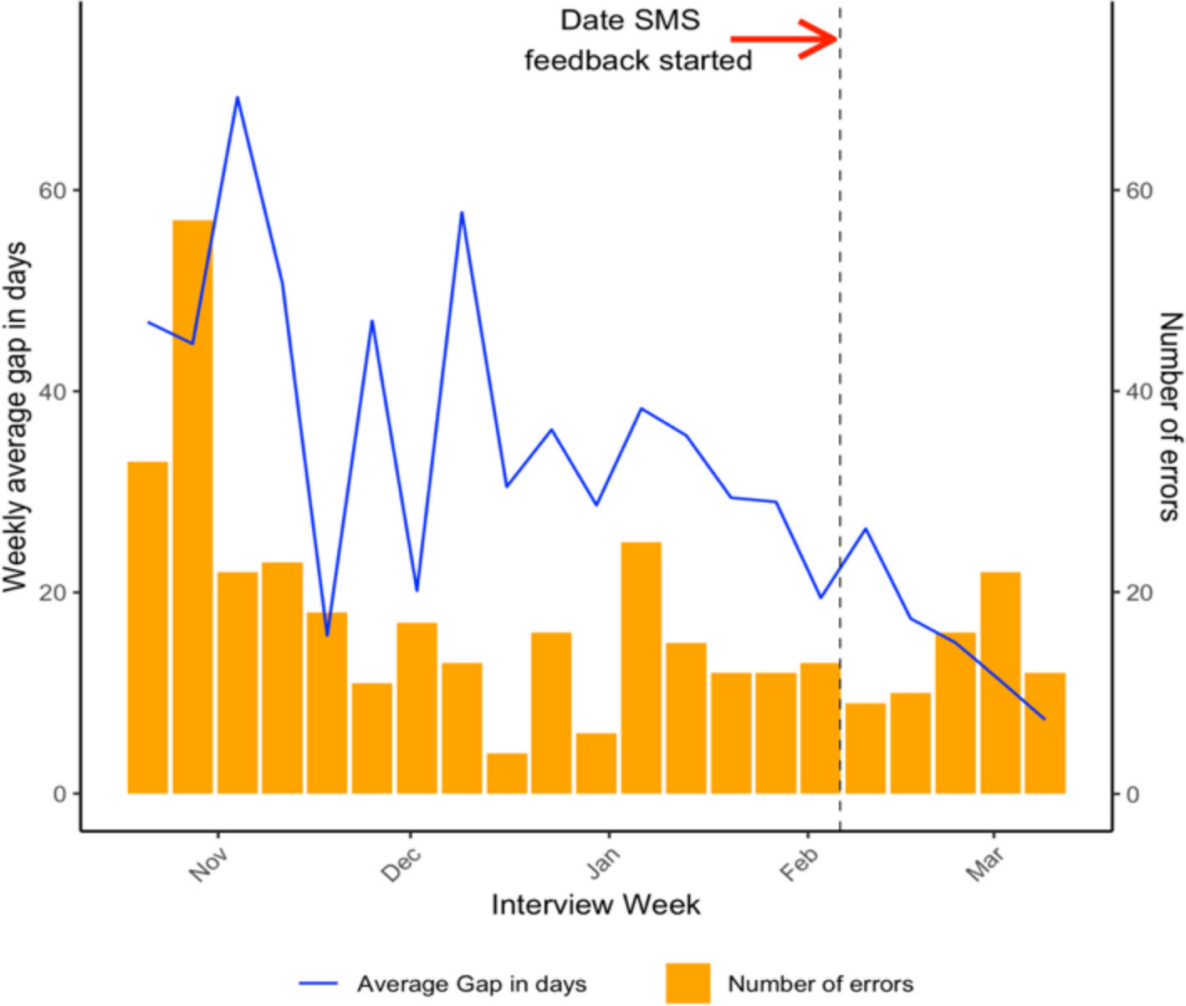
The weekly average number of days between interview date and date of error resolution as well as the weekly average number of errors decreased over time. SMS, short messaging service.

When examining the gap between interview date and error resolutions quantitatively, we see that both the weekly average number of days of gap as well as the weekly average number of errors per week decrease over time (figure 2). A limitation of the study includes the late initiation of this SMS feedback programme: it was implemented towards the end of the study when the gap between interview date and error resolution date was constrained by the end date of data collection. Additionally, by the end of the study, fewer issues were identified because the field staff was cognizant of the potential errors and more practiced in avoiding those errors. At the beginning of the study, the field staff was still becoming comfortable with the survey tool itself and the logistics of data collection; thus, the number of errors was higher. Additionally, because data collection had just started and there were weeks to resolve errors, error resolution was a slower process.

### Modality

The field team said that SMS was a good medium in general, but if given the choice, would have preferred WhatsApp. While they noted that SMS would be best in areas with limited internet connectivity, they mentioned that the field accommodations had sufficiently strong internet connectivity to support the use of WhatsApp. We did not have issues with SMS inboxes being full, but this is another technical constraint to consider when selecting the modality. Additionally, respondents said they preferred group messages over individual messages. Because the error resolution required coordination and communication among the team, supervisors and coordinators copied and pasted the error SMSs into their WhatsApp group. The survey coordinator was also part of this WhatsApp group and received the resolutions to each error message on this group and then transferred the correction to the master Google spreadsheet. Additionally, one supervisor initially thought that the SMS messages were a two-way system where they could send in the correction to an error message as a reply. This is another modality we could explore in future surveys where we have a greater number of errors.

R: So, it’s better if it is sent in a group. It could be a message in a group or SMS or WhatsApp anything. Other people also get a chance to rectify their mistakes. If a mistake is coming for one person, then others also feel that he could also commit that mistake there, so he should take more care over here. (COR_02)

R: I have seen it myself at many places. People don’t see text messages. Some have 500 unread messages while some have 100. But, they don't have any unread WhatsApp messages because they see it all the time or every second. Today’s generation is opening WhatsApp at every second. (SUP_03)

### Recipients of system

In terms of who should receive messages, coordinators said that everyone, including enumerators, should receive messages because then they can take responsibility either personally or coordinate through a group message. On the other hand, supervisors and enumerators were split on if enumerators should also receive messages. One supervisor felt odd bothering the female enumerators late at night and preferred that the enumerators receive the messages directly; whereas, another supervisor felt sending messages out to everyone would decrease their value. A third supervisor said resolving mistakes was their responsibility, and enumerators should not be getting the messages. One enumerator felt that getting a personal message would be better than a supervisor informing their subteam of a mistake; whereas, another enumerator felt learning about others’ mistakes was a learning opportunity.

R: This [SMS] should be limited to the coordinator and the supervisor, if many people are engaged then the value of it decreases, the value of that SMS will be lost if the enumerator is also joined…. (SUP_02)

R4: Your method is best that in my phone, I am receiving SMS only about my errors and no one else is receiving that message, whether SMS or WhatsApp. But if Supervisor sends that message to everyone, then it will not be good, in such case it would be best that supervisor personally tells about our mistake”. (FGD_ENUM)

## Future use and conclusions

Overall reactions to the SMS feedback system were encouraging. The feedback system has helped the field team resolve errors in a timely manner, thereby improving the data quality, and reduced their workload as compared with manual notification of errors. Most field staff had not been exposed to such a SMS feedback system and were enthusiastic about having such a system in place for future surveys where it would be implemented earlier in the project with a group message modality preferably on WhatsApp. They felt the regular feedback helped them improve their interviews and would have improved even more with an earlier implementation of the SMS notifications. Moreover, some training or briefing of the error resolution system could have streamlined the introduction and process. We recognise that this study of the SMS system was limited by the fact that the study team was researching its own enumerators and field staff and that there was no comparator to this system beyond what was occurring before it was introduced. Given the importance of household surveys in collecting health information and the widespread use of technology across most areas, this pilot of a SMS notification programme as a means to improve QA could be further studied and assessed as well as scaled up for future surveys keeping in mind aspects such as field staff dynamics, modality preferences, technical constraints, and specific survey error types.

## Data Availability

Data are available upon request from the Study PI (Dr. Amnesty LeFevre, aelefevre@gmail.com).
